# Membrane lipid modulations by methyl-β-cyclodextrin uncouple the *Drosophila* light-activated phospholipase C from TRP and TRPL channel gating

**DOI:** 10.1016/j.jbc.2023.105484

**Published:** 2023-11-21

**Authors:** Rita Gutorov, Ben Katz, Maximilian Peters, Baruch Minke

**Affiliations:** Faculty of Medicine, Institute for Medical Research Israel-Canada (IMRIC), Edmond and Lily Safra Center for Brain Sciences (ELSC), The Hebrew University, Jerusalem, Israel

**Keywords:** TRP channel, *Drosophila*, PLC, ergosterol, MβCD

## Abstract

Sterols are hydrophobic molecules, known to cluster signaling membrane-proteins in lipid rafts, while methyl-β-cyclodextrin (MβCD) has been a major tool for modulating membrane-sterol content for studying its effect on membrane proteins, including the transient receptor potential (TRP) channels. The Drosophila light-sensitive TRP channels are activated downstream of a G-protein–coupled phospholipase Cβ (PLC) cascade. In phototransduction, PLC is an enzyme that hydrolyzes phosphatidylinositol 4,5-bisphosphate (PIP2) generating diacylglycerol, inositol-tris-phosphate, and protons, leading to TRP and TRP-like (TRPL) channel openings. Here, we studied the effects of MβCD on Drosophila phototransduction using electrophysiology while fluorescently monitoring PIP2 hydrolysis, aiming to examine the effects of sterol modulation on PIP2 hydrolysis and the ensuing light-response in the native system. Incubation of photoreceptor cells with MβCD dramatically reduced the amplitude and kinetics of the TRP/TRPL-mediated light response. MβCD also suppressed PLC-dependent TRP/TRPL constitutive channel activity in the dark induced by mitochondrial uncouplers, but PLC-independent activation of the channels by linoleic acid was not affected. Furthermore, MβCD suppressed a constitutively active TRP mutant–channel, trpP365, suggesting that TRP channel activity is a target of MβCD action. Importantly, whole-cell voltage-clamp measurements from photoreceptors and simultaneously monitored PIP2-hydrolysis by translocation of fluorescently tagged Tubby protein domain, from the plasma membrane to the cytosol, revealed that MβCD virtually abolished the light response when having little effect on the light-activated PLC. Together, MβCD uncoupled TRP/TRPL channel gating from light-activated PLC and PIP2-hydrolysis suggesting the involvement of distinct nanoscopic lipid domains such as lipid rafts and PIP2 clusters in TRP/TRPL channel gating.

Cyclodextrins are a family of cyclic oligosaccharides, consisting of glucose subunits arranged as macrocyclic rings. Chemically, the interior part of cyclodextrins is hydrophobic, while the exterior part is hydrophilic promoting formation of complexes with hydrophobic compounds. A common method for modulating sterol level in the plasma membrane is by incubation of cells with methyl-β-cyclodextrin (MβCD), a cyclic oligosaccharide consisting of a macrocyclic ring of seven glucose subunits joined by α-1,4 glycosidic bonds. MβCD has preferential binding toward sterols compared to phospholipids, allowing sequestration or enrichment of sterols of living cells membranes (1). Sterol-saturated MβCD is efficient as sterol donor, enabling sterol enrichment by ∼30% to ∼3-fold (2, 3). Incubation of cells with high concentration of "empty" MβCD (5–10 mM, designated MβCD) for hours (>2 h) can reduce the total cellular sterol levels by 80 to 90% (2, 4). Importantly, sterol sequestration leads to disassociation of proteins from lipid rafts (5, 6, 7) and decrease clustering of raft-associated molecules (8). In contrast, sterol sequestration has virtually no effect on phosphatidylinositol 4,5-bisphosphate (PIP_2_) clusters (PIP_2_ microdomains) in the inner-leaflet plasma membrane of cells, which are physically separated from sterol-containing lipid rafts (9, 10, 11).

*Drosophila* phototransduction is a G-protein–coupled and phospholipase C (PLC)-mediated cascade, with transient receptor potential (TRP) and TRP-like (TRPL) as the transducer channels. Hydrolysis of PIP_2_ by PLC result in generation of diacylglycerol (DAG), reduction of PIP_2_ and generation of protons. All or some of these events are crucial for the physiological activation of TRP and TRPL channels and the generation of the light-induced current (LIC). However, the mechanism of channel gating is still under debate ((12, 13, 14, 15, 16, 17, 18), reviewed in (13, 19, 20)). Mutations in proteins of the phosphoinositide cycle, which mediate conversion of the PLC product DAG back to PIP_2_ have been shown to induce light-dependent and light-independent photoreceptor degeneration and affect the TRP and TRPL channel activity (21). Application of poly unsaturated fatty acids (PUFAs) robustly activated the TRP/TRPL channels in the dark in *Drosophila* photoreceptors (22) in a PLC-independent manner (23). ATP depletion also activated the TRP/TRPL channels in the dark (24), but in a PLC-dependent manner (25). The activity of *Drosophila* TRPL channels expressed in tissue culture cells was shown to be suppressed by incubation with MβCD (26). However, localizing the effect of sterol reduction on the phosphoinositide cascade that is natively expressed in these cells was not investigated.

Unlike vertebrates, flies are unable to synthesize sterols (auxotroph) and receive this essential lipid compound from their yeasts diet in the form of ergosterol (27). Dietary restriction of ergosterol intake of flies resulted in disruptive association of phototransduction signaling components with detergent-resistant membrane (DRM) lipid raft fractions (28). These signaling complexes included the scaffold protein inactivation-nο-afterpotential D (INAD), which binds major phototransduction components such as PLC and TRP. Importantly, these signaling proteins were found to be associated, in a light-dependent manner, with DRM lipid rafts domain. Hence, reduction of ergosterol, considered to be a key component of lipid rafts in *Drosophila*, resulted in a loss of INAD-signaling complexes associated with DRM lipid rafts fractions (28). However, the effects of ergosterol reduction by dietary restriction on the light response were not examined in this comprehensive biochemical study.

In the present study we extended the previous reports, which reduced ergosterol levels in *Drosophila* photoreceptors by dietary manipulations, and examined, for the first time, the effect of ergosterol reduction by MβCD on PIP_2_ hydrolysis, when measured together with the physiological response to light. Accordingly, we used whole-cell voltage-clamp measurements from photoreceptor cells and simultaneously monitored PIP_2_ hydrolysis by translocation of fluorescently tagged lipid-binding Tubby protein domain, from the plasma membrane to the cytosol. These measurements revealed that incubation with MβCD virtually abolished the light response while having only little effect on the light activated PIP_2_ hydrolysis by PLC. Furthermore, MβCD suppressed a constitutively active TRP mutant–channel, *trp*^*P365*^, suggesting that TRP channel activity is a target of MβCD action. Together, MβCD uncoupled TRP/TRPL channel's gating from light-activated PLC and PIP_2_ hydrolysis, suggesting involvement of nanoscopic lipid domains such as lipid rafts and PIP_2_ clusters in TRP/TRPL channel's gating.

## Results

### MβCD suppressed the LIC of the null mutant flies *trpl*^*302*^ (expressing only the TRP channel) and *trp*^*P343*^ (expressing only the TRPL channel)

**Figure 1:**
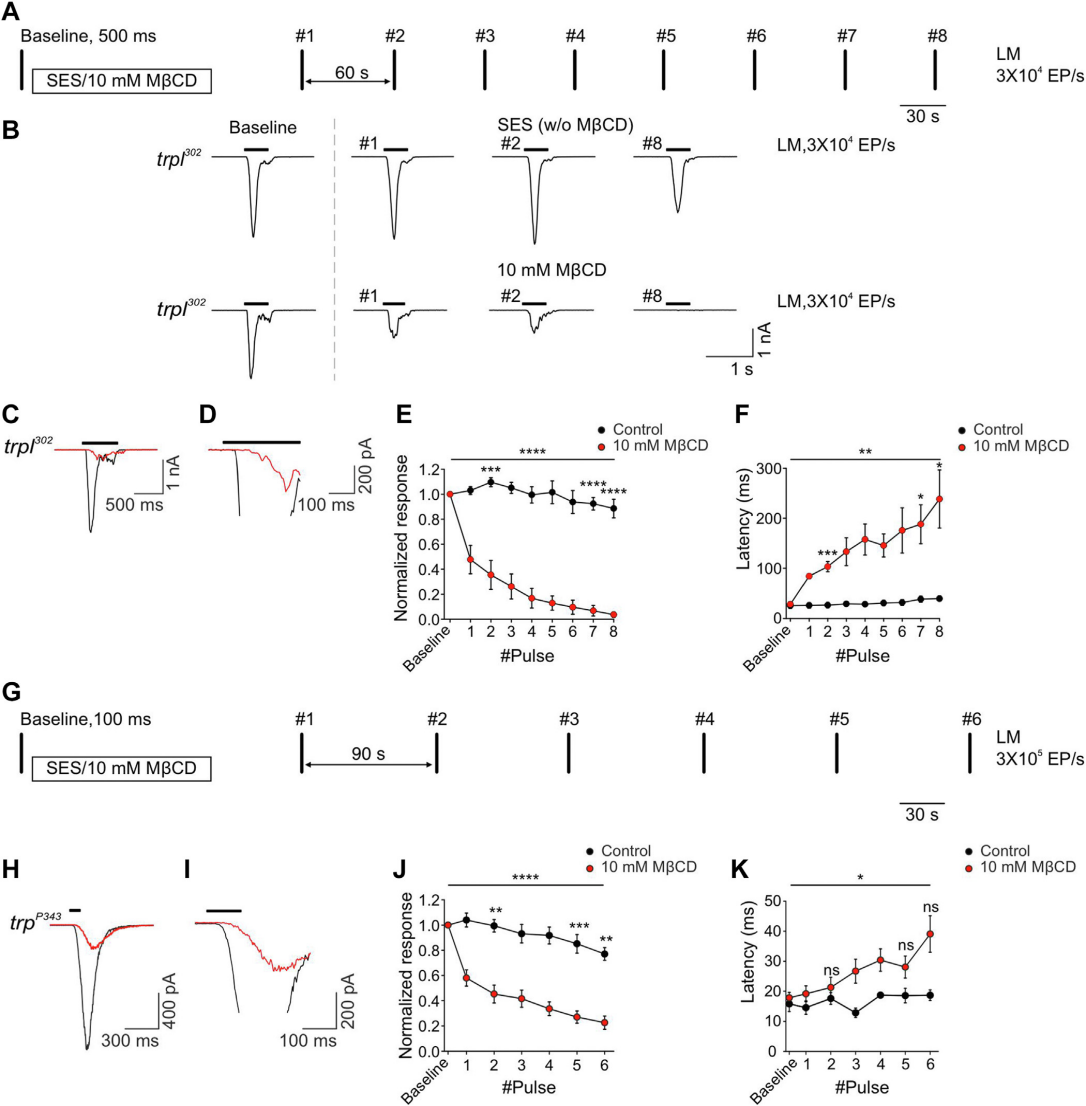
**MβCD strongly suppressed the light-induced current arising from activation of the TRP and TRPL channels.***A*, a schematic presentation of the experimental protocol. Each *vertical line* represents a given *orange light pulse* of 500 ms duration (light monitor, LM), at an intensity of 3 × 10^4^ effective photons/s. The first light pulse was given after establishing the whole-cell configuration, constituting a control response. Following the control pulse, the standard extracellular solution (SES) or 10 mM MβCD was perfused into the bath for 2 min until the bath solution was virtually replaced. One minute after the perfusion was stopped, a train of eight light pulses was applied, with 1 min intervals between the light pulses. *B*, representative traces of whole-cell voltage-clamp recordings recorded from *dark* adapted isolated ommatidia of the *trpl*^*302*^ mutant (expressing the TRP channels), showing the light-induced currents (LIC) in response to the control, first, second, and eighth light pulses, in the presence of either SES (*top*) or 10 mM MβCD (*bottom*). *C* and *D*, representative LICs recorded from *dark* adapted photoreceptor cells of the *trpl*^*302*^ mutant in response to the fourth light pulse, in the presence of SES (*black*) or 10 mM MβCD (*red*). D is a magnified LIC (similar to *trace C*), depicting the latency of the LIC. The *black horizontal line* represents the light monitor (LM) in all traces. *E*, graphs presenting the normalized peak amplitude of the responses to repetitive *orange light pulses* as a function of time, recorded from *trpl*^*302*^ mutant flies, in the presence (*red*, n = 6) and absence (*black*, n = 6) of 10 mM MβCD. Each light response was normalized to the control LIC for each fly. *Asterisks* indicate statistical significance for two-way ANOVA test (*p* < 0.0001) with Holm-Sidak’s correction for multiple comparisons for three representative pulses: the second pulse after the perfusion (*p* = 0.001) and the last two pulses (*p* < 0.0001). Each point represents the average of all flies and the error bars are the SEM. *F*, graphs presenting the latency from the light onset to the beginning of the light response to repetitive *orange light* as a function of time, recorded from *trpl*^*302*^ mutant flies, in the presence (*red*, n = 6) and absence (*black*, n = 6) of 10 mM MβCD. *Asterisks* indicate statistical significance for two-way ANOVA test (*p* = 0.0033) with Holm-Sidak’s correction for multiple comparisons for three representative pulses: the second pulse after the perfusion (*p* = 0.0033) and the last two pulses (*p* = 0.0245). Each point represents the average of all flies and the error bars are the SEM. *G*, a schematic presentation of the experimental protocol. Each *vertical line* represents a given *orange light pulse* of 100 ms duration (light monitor, LM), at an intensity of 3 ×10^5^ effective photons/s. The first light pulse was given after establishing the whole-cell configuration, constituting a control response. Following the control pulse, the standard extracellular solution (SES) or 10 mM MβCD was perfused into the bath for 2 min until the bath solution was virtually replaced. One minute after the perfusion was stopped, a train of eight light pulses was applied, with 90 s intervals between the light pulses. *H*, representative traces of whole cell voltage-clamp recordings obtained from *dark* adapted isolated-ommatidia of the *trp*^*P343*^ mutant (expressing the TRPL channels), showing the LIC in response to *orange light* (as indicated by *horizontal bar*) after 4 min of incubation in the presence of SES (*black*) or 10 mM MβCD (*red*). *I*, magnified LIC of H, which depicts the latency following the light pulse. *J*, graphs presenting the normalized peak amplitude of the responses to repetitive *orange light pulses* as a function of time, recorded from *trp*^*P343*^ mutant flies, in the presence (*red*, n = 6) and absence (*black*, n = 6) of 10 mM MβCD. Each light response was normalized to the control LIC for each fly. *Asterisks* indicate statistical significance for two-way ANOVA test (*p* < 0.0001) with Holm-Sidak’s correction for multiple comparisons for three representative pulses: the second pulse after the perfusion (*p* = 0.0012) and the last two pulses (*p* = 0.0004 and *p* = 0.0011). Each point represents the average of all flies and the error bars are the SEM. *K*, graphs presenting the latency from the light onset to the beginning of the light response to repetitive *orange light* as a function of time, recorded from *trp*^*P343*^ mutant flies, in the presence (*red*, n = 6) and absence (*black*, n = 6) of 10 mM MβCD. *Asterisks* indicate statistical significance for two-way ANOVA test (*p* = 0.0187) with Holm-Sidak’s correction for multiple comparisons for three representative pulses: the second pulse after the perfusion and the last two pulses (*p* > 0.05). Each point represents the average of all flies and the error bars are the SEM. MβCD, methyl-β-cyclodextrin; TRP, transient receptor potential; TRPL, TRP-like.

Following the finding that application of MβCD suppresses the activity of the *Drosophila* TRPL channel expressed in tissue culture cells (26), we thought of examining the effects of MβCD on the *Drosophila* TRP and TRPL channels in the native photoreceptor cells. First, we examined the effect of MβCD (10 mM), on the light response of the *trpl*^*302*^ null mutant (in which the LIC is composed only of TRP channels, Fig. 1, *A*–*F*) and *trp*^*P343*^ null mutant flies (in which the LIC is composed of only of TRPL channels, Fig. 1, *G*–*K*). Accordingly, the LIC in response to a train of brief intense orange light pulses, separated by dark intervals (60 s for *trpl*^*302*^ and 90 s for *trp*^*P343*^) were measured using the whole-cell voltage-clamp technique (*trpl*^*302*^ mutant, Fig. 1, *A* and *B* and *trp*^*P343*^ mutant Fig. 1, *G*–*I*), in the presence and absence of MβCD. The peak amplitude and latency (the time from light onset to the beginning of the response) of the LICs remained relatively constant under incubation with standard extracellular solution (SES) of both the *trpl*^*302*^ and *trp*^*P343*^ mutants (Fig. 1, *B*, *E*, and *J*, respectively), with only slight decrease in response amplitude and increase in response latency after ∼9 min (#8 light pulse for *trpl*^*302*^, #5 light pulse for *trp*^*P343*^, Fig. 1, *F* and *K*, respectively). Interestingly, incubation of the photoreceptor cells of the *trpl*^*302*^ with MβCD significantly reduced the amplitude and increased the latency of the LIC (Fig. 1, *E* and *F*), while incubation of the photoreceptor cells of the *trp*^*P343*^ with MβCD significantly reduced the light response amplitude but increased the response latency only to a small extent (Fig. 1, *J* and *K*).

In summary, incubation of photoreceptor cells from *trpl*^*302*^ (expressing only the TRP channel) and *trp*^*P343*^ flies (expressing only the TRPL channel) with MβCD decreased the amplitude and slowed the kinetics of the LIC.

### MβCD reduced the frequency and to a lesser extent the amplitude of the responses to single photons

**Figure 2:**
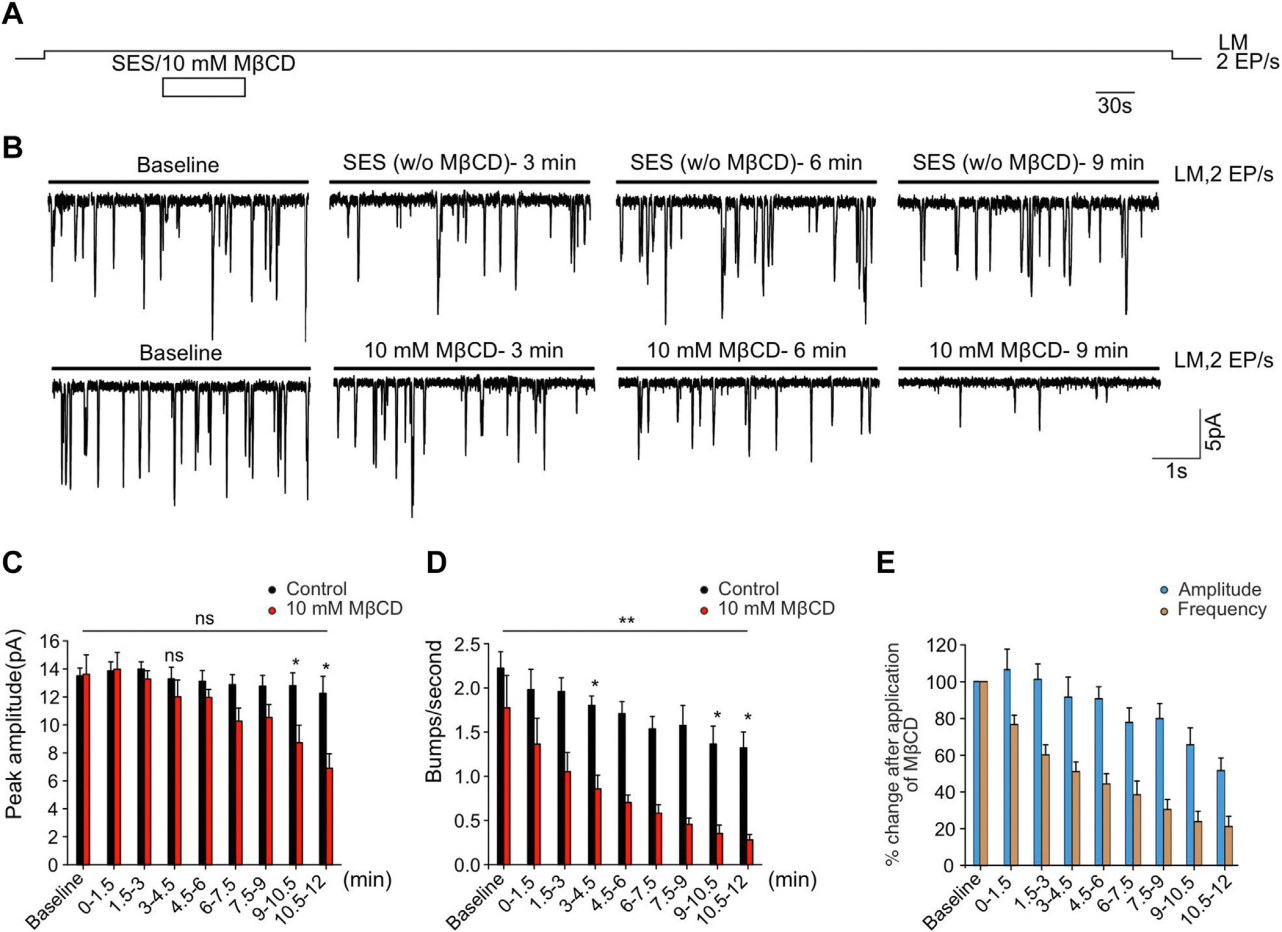
**MβCD reduced the frequency and to a lesser extent the amplitude of the responses to single photons.***A*, a schematic presentation of the experimental protocol. A continuous *dim light* (two effective photon/s) was given after the whole-cell configuration was established, for a duration of 14.5 min. Ninety seconds after the beginning of illumination, either SES or 10 mM MβCD was perfused into the bath for 1 min. *B*, representative traces showing generation of quantum bumps recorded from the *trpl*^*302*^ mutant in response to the *continuous dim orange light* before perfusion (control) and after 3, 6, 9 min of perfusion with either SES (*top*) or MβCD (*bottom*). *C*, histogram displaying the average peak amplitude of the quantum bumps of the *trpl*^*302*^ mutant as a function of time. Control peak amplitude was measured during 90 s before perfusion onset and the time intervals are the duration of incubation with either SES (*black*, n = 6) or MβCD (*red*, n = 6). *Asterisks* indicate statistical significance for two-way ANOVA test (*p* > 0.05) with Holm-Sidak’s correction for multiple comparisons for three representative time points: 3 to 4.5 (*p* > 0.05), 9 to 10.5 (*p* = 0.0368), and 10.5 to 12 (*p* = 0.0409) minutes after the perfusion. Each point represents the average of all flies and the error bars are the SEM. *D*, histogram displaying the quantum bump frequency of the *trpl*^*302*^ mutant as a function of time. Control measurement was obtained 90 s before perfusion onset, and the time intervals are the duration of incubation with either SES (*black*, n = 6) or MβCD (*red*, n = 6). *Asterisks* indicate statistical significance for two-way ANOVA test (*p* = 0.0012) with Holm-Sidak’s correction for multiple comparisons for three representative time points: 3 to 4.5 (*p* = 0.0251), 9 to 10.5 (*p* = 0.0147), and 10.5 to 12 (*p* = 0.0215) minutes after the perfusion. Each point represents the average of all flies and the error bars are the SEM. *E*, histogram presenting the reduction in normalized mean bump amplitude (*blue columns*) and mean rate of occurrence (*orange columns*) relative to control, (% change after application of MβCD) reveals a larger reduction in bump rate as than bump amplitude at each indicated time interval following application of MβCD. Calculated from the data of the histograms of (*C* and *D*), Error bars are the SEM. MβCD, methyl-β-cyclodextrin; SES, standard extracellular solution.

*Drosophila* photoreceptor cells have reached the ultimate sensitivity to light, by responding to absorption of single photons with discrete voltage (or current) change called quantum bump (29, 30). In WT flies, a quantum bump is the result of synchronized activation of several TRP and TRPL light-sensitive channels in a single microvillus (31). Quantum bump of WT and the *trpl*^*302*^ mutant flies (expressing only the TRP channel) display large amplitude (∼8–14 pA) under standard condition (32, 33) that can be readily measured and analyzed. However, quantum bumps of *trp*^*P343*^ mutant flies (expressing only the TRPL channel) are small in amplitude (∼3 pA), making measurement and analysis extremely challenging and therefore bump analysis was performed only on *trpl*^*302*^ mutant photoreceptors. Since the macroscopic LIC constitutes a summation of quantum bumps (34, 35), changes in bumps amplitude, frequency, waveform, or latency distribution affect the macroscopic light response. To analyze the effect of MβCD on bump parameters, we recorded single photon responses under dim light illumination from the *trpl*^*302*^ mutant flies (expressing TRP channels) in the presence and absence of MβCD. Bumps recordings and analysis under standard condition from photoreceptors of *trpl*^*302*^ mutant flies revealed that the bump amplitude was relatively stable (∼13 pA, see (36)) during 15 min of recording (Fig. 2*C*, control). Bump frequency under standard condition started at ∼2 bumps/s and decreased by ∼25% reaching ∼1.5 bumps/s after 15 min of recording under constant continuous dim light (Fig. 2*D*, MβCD, control (33)). Incubation of the photoreceptors with MβCD caused a time-dependent decrease in bump frequency reaching a reduction of ∼85% after ∼10 min (Fig. 2, *B* and *D*). In contrast, incubation of the photoreceptors with MβCD had relatively small effect on the bump's amplitude, which began showing reduced amplitudes only after ∼7 min and reaching a ∼50% reduction after ∼10 min (Fig. 2, *B* and *C*, MβCD). At the 10.5 to 12 min time point of incubation with 10 mM MβCD, the mean bump amplitude was reduced to ∼7 pA and mean bump frequency to ∼0.27 bumps per second. To emphasize the differential effect of MβCD on bump amplitude relative to bump frequency, a histogram is shown in Figure 2*E* presenting the reduction in the normalized mean bump amplitude (blue columns) and mean bump frequency (orange columns) relative to their control, (% difference). The histogram shows a significant larger reduction in bump frequency as than the reduction in bump amplitude following application of MβCD (Fig. 2*E*, see the implications in the Discussion).

### MβCD suppressed channel activity of the TRP and TRPL channels induced by metabolic inhibition in the dark

**Figure 3:**
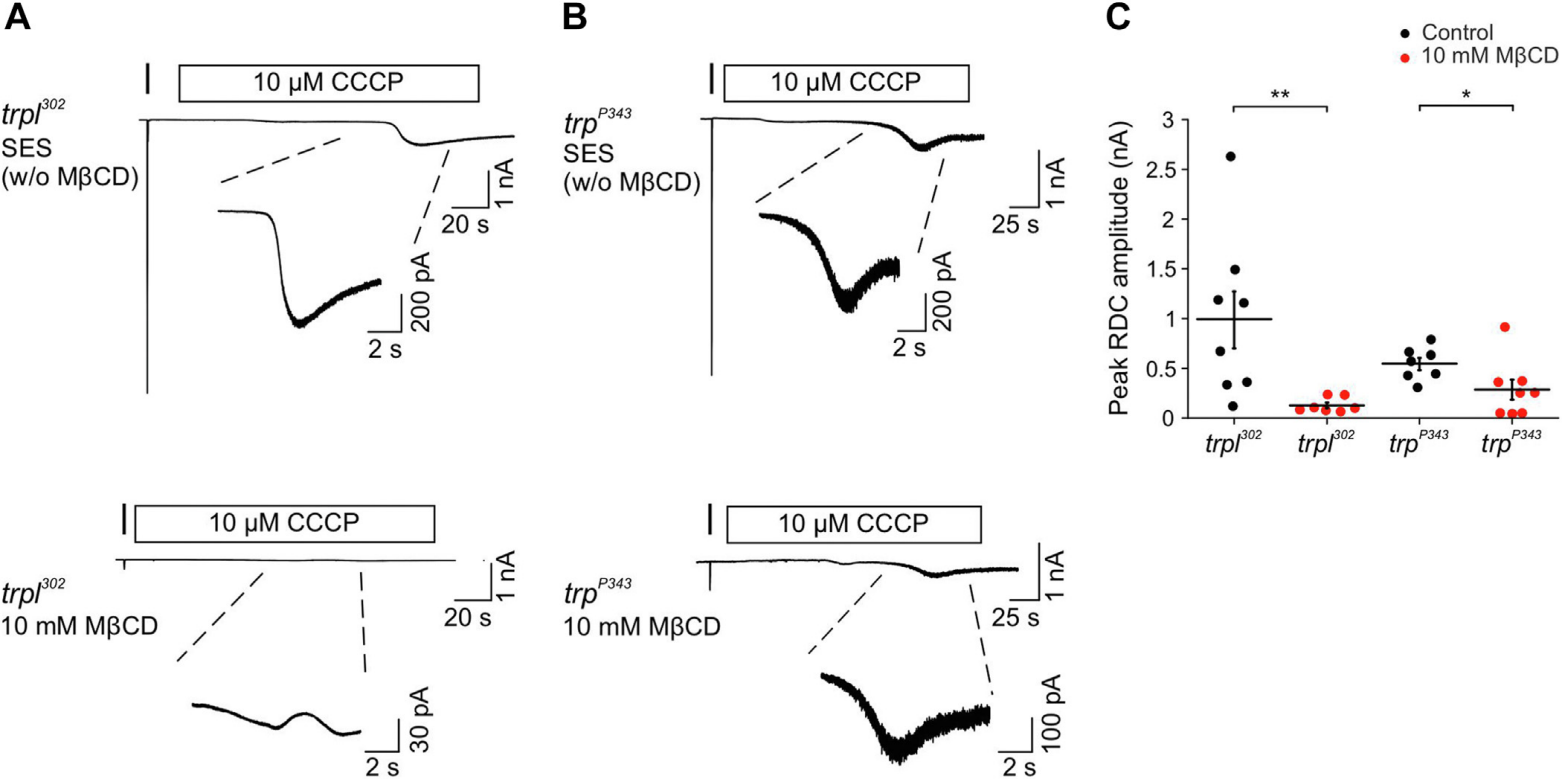
**MβCD strongly suppressed TRP and TRPL channels activity induced by metabolic inhibition in the dark.***A* and *B*, *top*: control whole-cell patch clamp recordings displaying TRP and TRPL channels activity induced by application of the mitochondrial uncoupler CCCP (10 μM, *rectangles*) to isolated ommatidia of *trpl*^*302*^ (*A*) and *trp*^*P343*^ (*B*) mutant flies in the *dark*. A *short orange test light pulse* is represented by the *vertical line*. Note the larger channel noise of the *trp*^*P343*^ current that arises from the ∼10-fold larger single-channel conductance of TRPL relative to TRP channels (37). *Bottom:* whole-cell patch clamp recordings from *trpl*^*302*^ (*A*) and *trp*^*P343*^ (*B*) mutant flies displaying activated channels following application of 10 μM CCCP (*rectangles*) in the presence of 10 mM MβCD in the *dark*. *C*, *scatter plot* showing the peak amplitude of the current resulting from channel activation by 10 μM CCCP-inducing metabolic inhibition of *trp*^*302*^ and *trp*^*P343*^ mutant photoreceptors (peak RDC amplitude), in the presence (*red*, n_*trpl302*_ = 7 n_*trpP343*_ = 8) or absence (*black*, n_*trpl302*_ = 8 n_*trpP343*_ = 7) of MβCD. *Asterisks* indicate statistical significance for two-tailed Mann-Whitney *U* test (∗*p* = 0.0277, ∗∗*p* = 0.0012). CCCP, carbonyl cyanide m-chlorophenyl hydrazine; MβCD, methyl-β-cyclodextrin; RDC, rundown current; TRP, transient receptor potential; TRPL, TRP-like.

Previous studies have shown that it is possible to bypass the light activation of key signal transduction components of the phototransduction cascade and activate pharmacologically the TRP and TRPL channels in the dark *in vivo*. This method can assist in identifying molecular components of the phototransduction cascade, which are sensitive to pharmacological agents. One such way is to induce metabolic inhibition by anoxia *in vivo* (24) or by depleting ATP from the photoreceptor cell *ex vivo*, using specific mitochondrial uncouplers such as carbonyl cyanide m-chlorophenyl hydrazine (CCCP, (24)). Under conditions of ATP depletion, dark activation of the TRP/TRPL channels is not manifested by synchronous channel activation like channel activation by the absorbed photons (quantum bumps) but rather by continuous noisy slow inward current called rundown current (RDC), which is composed of channel noise that arises directly from non-synchronous activation of the TRP (Fig. 3*A* top, Fig. 3*C* control, left) and TRPL channels (Fig. 3*B*, top, Fig. 3*C* control, right (24, 37)).

A suggested explanation for the ATP depletion–mediated dark channel activation came from studies of flies mutated in the retinal degeneration A (*rdgA*) gene encoding for DAG kinase (38). The *rdgA* mutant fly shows light-independent retinal degeneration (39) and dark activity of the TRP/TRPL channels (21) similar to that observed, following TRP and TRPL channel activation by mitochondrial uncouplers (24, 40). Therefore, it was suggested that cellular ATP depletion promotes TRP and TRPL channel opening as a result of DAG accumulation caused by the inhibition of DAG kinase, either directly by the *rdgA* mutation or indirectly by ATP depletion. The presumed accumulation of DAG in the dark in the *rdgA* mutants or in ATP-depleted photoreceptors was suggested to arise from a small basal (leak) activity of PLC (41). Hence, activation of the TRP and TRPL channels by ATP depletion does not involve signaling proteins upstream of PLC.

In order to identify the molecular component in the signal transduction cascade, which is affected by incubation with MβCD, the response of *trpl*^*302*^ and *trp*^*P343*^ mutant photoreceptors to metabolic inhibition (using CCCP) in the dark was measured in the absence and presence of MβCD. MβCD strongly suppressed both the TRP- and TRPL-dependent currents (TRP: Fig. 3*A*, bottom, Fig. 3*C*, red, left; TRPL: Fig. 3*B*, bottom, Fig. 3*C*, red, right) that were induced by CCCP application in the dark. Since the CCCP-induced TRP and TRPL currents depend on a leak of PLC activity in the dark (42), the results suggest that at least some of the effect of MβCD is either at the PLC level or downstream of PLC activation (*e.g.*, at the level of the light activated channels).

### Light-activated PLC hydrolyzing activity was maintained in the presence of MβCD that virtually abolished the LIC

**Figure 4:**
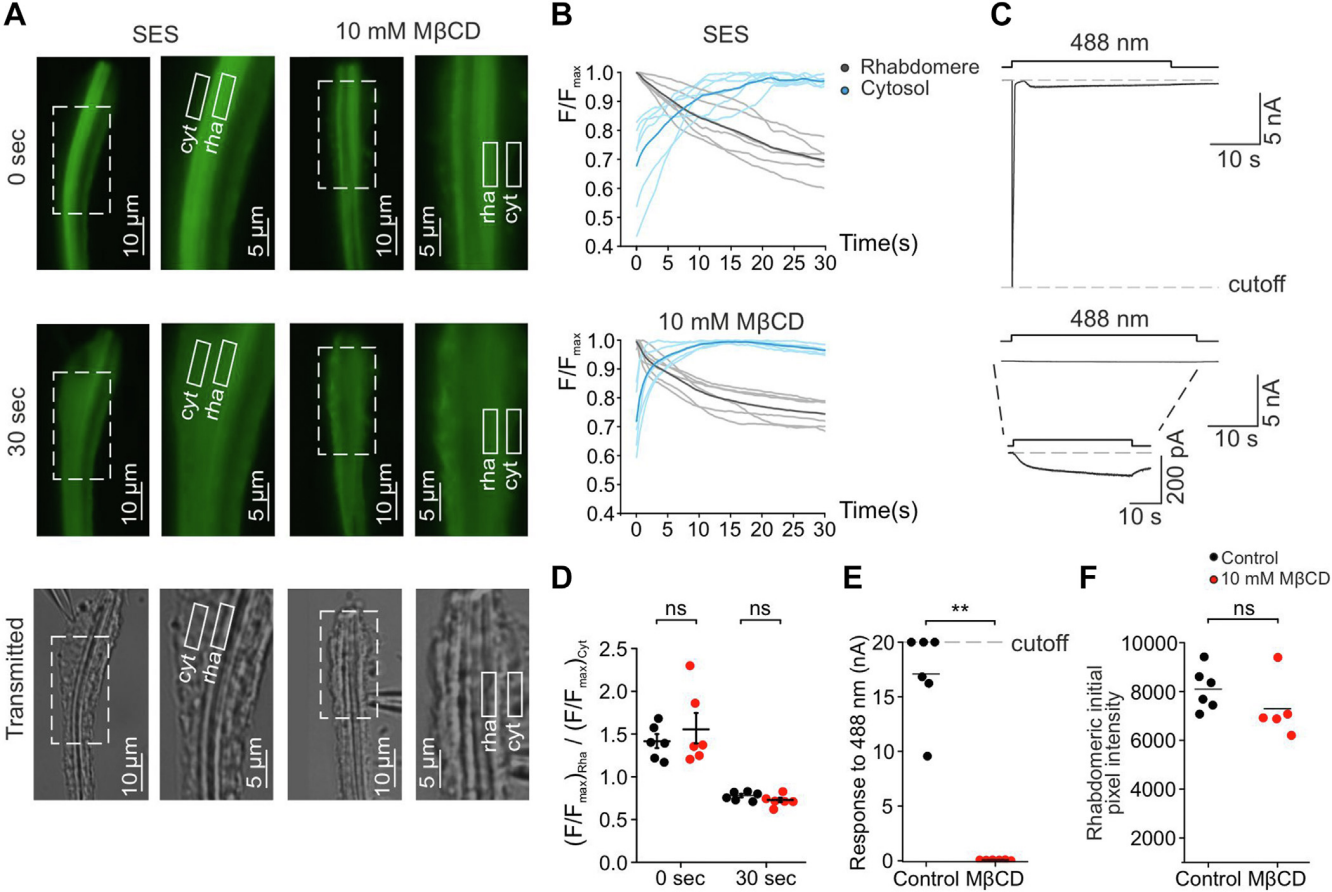
**MβCD did not inhibit light-activated PLC-hydrolyzing activity during strong suppression of the LIC: Simultaneous recordings of the LIC and light-induced PIP**_**2**_**hydrolysis as monitored by Tb**^**R332H**^**-YFP fluorescence translocation from the rhabdomere to the cytosol.***A*, *top* and *middle*: fluorescent images from initially dark-adapted dissociated ommatidium expressing Tb^R332H^, immediately at the onset of *intense blue excitation light* (*top*) and 30 s later (*middle*). Fluorescence was initially strongest in the rhabdomeres, but rapidly translocated to cell body and plasma membrane (*white rectangulars*). The ommatidium of the *left pair* was perfused with SES containing 1.5 mM Ca^2+^ serving as a control. MβCD (10 mM) was added to the SES of the same ommatidium (*right pair*). *Bottom*: photographic images from initially dark adapted dissociated ommatidium expressing Tb^R332H^ in the presence or absence of MβCD. The right transmission picture of each photographic pair (*i.e.*, SES or 10 mM MβCD) is an *enlarged image* of the area represented by the *dashed rectangular* (*left of each pair*). The rhabdomere (rha) and cytosol (cyt) are indicated by *white rectangular*. *B*, the normalized florescence ratio was measured over time in the areas indicated by *solid white rectangles* of the rhabdomere (*black*) and the cytosol (*blue*), in the presence (*bottom*) or absence (*top*) of MβCD. The *bold black* and *blue lines* represent the mean ratio (n = 6). Note the faster kinetics in the presence of MβCD. *C*, whole-cell voltage-clamp recordings from the dissociated ommatidium shown in (*A*), in response to a *long intense blue light pulse* (488 nm, *right*) in the absence (*top trace*) or presence of MβCD (*bottom trace* and a highly magnified inset). The *top recording* corresponds to the *left pair* of images in (*A*) and the *bottom recording* correspond to the *right pair* of images in (*A*). After establishing the whole-cell configuration, SES, or 10 mM MβCD was perfused into the bath for 2 min, until the bath solution was virtually replaced. In the presence of MβCD, 1 min after the perfusion was stopped, a series of orange (590 nm) light pulses were applied, with 1 min intervals between the pulses (not shown). Once the light response was virtually abolished, the florescence was measured by 30 s of *intense blue* (488 nm) *light excitation*. In the control experiments, 1 min after the perfusion was stopped, 1 to 3 *orange* (590 nm) *light pulses* were applied, with 1 min intervals between the pulses (not shown). The florescence was measured by 30 s of *intense blue* (488 nm) *excitation light*. *D*, a *scatter plot* showing the ratio of the normalized florescence measured from the rhabdomere and the cytosol immediately at the onset of the *blue excitation light* (0 s, *left*) and 30 s later (*right*), in the presence (*red*) or absence (*blue*) of MβCD (n = 6 for all experiments). Two-tailed Mann-Whitney *U* test was preformed (ns *p* > 0.05). *E*, a *scatter plot* showing the peak amplitude of the LICs measured during whole-cell voltage-clamp recordings from dissociated ommatidium in response to the *blue* (488 nm, *right*) *intense light* in the presence (*red*) or absence (*black*) of MβCD. Cut-off indicates saturation of the patch clamp amplifier. *Asterisks* indicate statistical significance for two-tailed Mann-Whitney *U* test (ns *p* > 0.05, ∗∗*p* = 0.0022). *F*, a *scatter plot* showing the ratio of the initial pixel intensity of the fluorescence measured from the rhabdomere immediately at the onset of the *blue excitation light*, in the presence (*red*) or absence (*blue*) of MβCD (n = 6 for all experiments). Two-tailed Mann-Whitney *U* test was preformed (ns *p* > 0.05). LIC, light-induced current; MβCD, methyl-β-cyclodextrin; PLC, phospholipase C; PIP_2_, phosphatidylinositol 4,5-bisphosphate; SES, standard extracellular solution; TRP, transient receptor potential; TRPL, TRP-like.

The activation of PLC by light is a crucial step in the physiological activation of TRP and TRPL channels (12, 13, 14, 16). To measure PLC activity in the native photoreceptor cells, we used transgenic *Drosophila* fly expressing a fluorescent-tagged probe that binds to PIP_2_. Specifically, we used a transgenic fly strain expressing Tb^R332H^-YFP, a mutated version of the fluorescently tagged lipid-binding Tubby protein domain under the *ninaE* (Rhodopsin 1) promotor. The Tubby protein (YFP-tagged) has a high affinity for PIP_2_ but very small affinity for inositol-tris-phosphate (43), while a point mutation of the same construct, Tb^R332H^, reports cellular PIP_2_ changes independently of inositol-tris-phosphate generation (44) and thus fits best our experiments. Upon PIP_2_ hydrolysis by intense blue light activation of PLC, the fluorescent Tb^R332H^-YFP probe translocate from the plasma membrane to the cytosol of the photoreceptor cells enabling an estimated measure of PLC hydrolyzing activity (45, 46). To follow PLC hydrolyzing activity, we monitored the subcellular localization of the Tb^R332H^-YFP probe in dissociated ommatidia, which were imaged with epi-fluorescence microscopy during whole-cell recordings. This experimental setup enabled performing simultaneous electrophysiological and fluorescent measurements, in order to examine the effect of MβCD on the LIC and PLC activity during intense illumination. In dark-adapted photoreceptors most of the Tb^R332H^-YFP fluorescence was localized to the rhabdomeres of the photoreceptors, a dense surface membrane composed of thousands of microvilli (Fig. 4*A*, 0 s, SES, Fig. 4*F* control, see Fig. S2). Following the onset of the intense blue (488 nm) illumination, the intensity of the fluorescent signal rapidly decreased in the rhabdomeres and increased in the region of the cell body (Fig. 4*A*, 30 s, SES, see Fig. S2), reflecting translocation of the Tb^R332H^-YFP probe as PIP_2_ was hydrolyzed. Hence, the translocation of Tb^R332H^-YFP indicated that PLC was activated during the blue light illumination. We repeated this experiment following incubation of the photoreceptors with 10 mM MβCD (Fig. 4*A*, MβCD, Fig. 4*F* MβCD, see Fig. S2). Under this condition, the LIC was virtually abolished during the intense blue illumination (Fig. 4*C*, top *versus* bottom, Fig. 4*E*, control *versus* MβCD) as expected from previous experiments, while a robust Tb^R332H^-YFP translocation was observed, similar to that measured under control conditions (Fig. 4*A*, 30 s, MβCD, see Fig. S2). The quantification of Tb^R332H^-YFP translocation as a function of time in control (Fig. 4*B*, SES) and under incubation with MβCD (Fig. 4*B*, MβCD) further indicated that there was Tb^R332H^-YFP translocation of similar magnitude in the presence or absence of MβCD. Moreover, the initial rhabdomeric fluorescence (indicated as pixel intensity) was similar in the presence and absence of MβCD (Fig. 4*F*). Thus, the results presented in Figure 4 indicated that blue light-induced PLC hydrolyzing activity took place during strong suppression of the TRP/TRPL channel activity by MβCD. Interestingly, the accelerated kinetics of Tb^R332H^-YFP translocation when the LIC was suppressed by MβCD (Fig. 4*B*) was a strong support for the reliability of the used methodology. This is because it has been shown that in *Drosophila* photoreceptors PLC activity reveals a bell-shaped dependence on cellular Ca^2+^ concentrations, showing inhibition at high Ca^2+^ concentrations (47). Accordingly, acceleration of PLC activity was previously observed at low intracellular Ca^2+^ (45, 46). Thus, it is expected that PLC activity will be accelerated when Ca^2+^ influx through the TRP channels is reduced due to suppression of the LIC by MβCD.

Together, the results indicated that while TRP/TRPL channels activity during prolonged intense blue light was strongly attenuated by incubation with MβCD, neither PLC activity nor the activity of the signaling proteins upstream to PLC (*i.e.*, the photopigment and G_q_) were suppressed. Therefore, the results suggest that MβCD exerted its effect downstream of PLC in the phototransduction cascade.

### MβCD suppressed constitutive activity of the pore-mutant TRP channel of the *trp*^*P365*^ mutant fly

**Figure 5:**
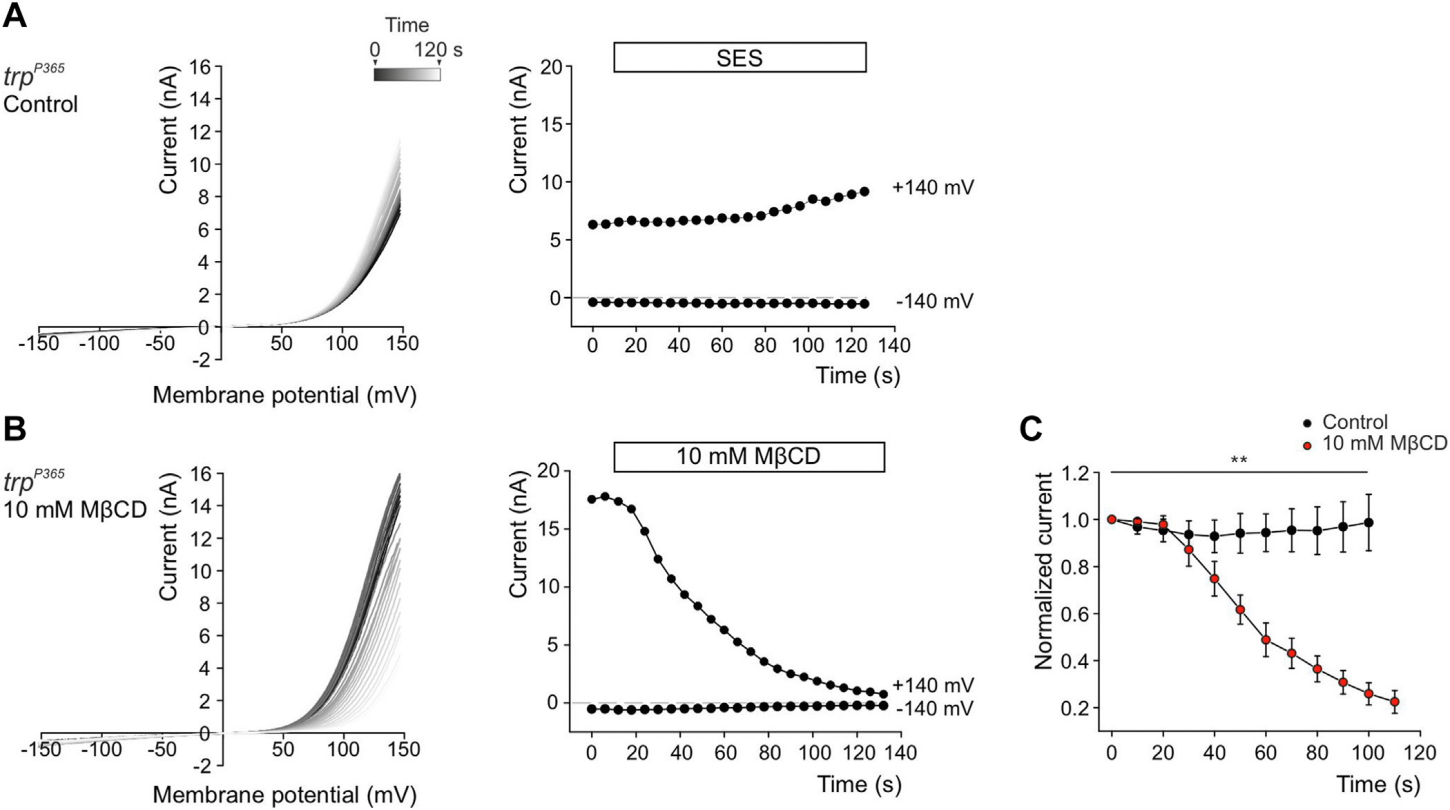
**MβCD strongly suppressed a constitutively active TRP-mutant channel (*trp***^***P365***^**) indicating that MβCD effect is at the channel gating level.***A* and *B*, *left*: representative family of current-voltage relationships (i-V curves) measured from isolated ommatidia of the *trp*^*P365*^ mutant, expressing constitutively active TRP channel, in the presence of SES (*A*) or 10 mM MβCD (*B*). The first i-V curve (time = 0, *rectangle*) is *black* and the gradient of *brighter color* indicates the progression of time in which each i-V curve was measured. *Right*: the current amplitude at ±140 mV holding voltages is plotted as a function of time in the presence of SES (*A*) and 10 mM MβCD (*B*). *C*, graphs presenting the normalized averaged currents of (*A*) (*right*) and (*B*) (*right*) as a function of time, in the presence (*red*, n = 6) and absence (*black*, n = 6) of 10 mM MβCD. Each data point was normalized relative to the control (SES) current for each fly. *Asterisks* indicate statistical significance for two-way ANOVA test (*p* = 0.002). Each point represents the average of all flies and the error bars are the SEM. Note that in ∼30% (3/11) of experiments MβCD did not suppress the *trp*^*P365*^ constitutive activity. MβCD, methyl-β-cyclodextrin; TRP, transient receptor potential; TRPL, TRP-like; SES, standard extracellular solution.

A random chemically induced mutagenesis of the *Drosophila* third chromosome yielded a mutant fly with extremely fast light-independent retinal degeneration. Genetic analysis of the mutant fly identified three mutations in the *trp* gene that was designated *trp*^*P365*^. Physiological analysis revealed that the mutations in the *trp*^*P365*^ mutant gave rise to constitutively active TRP channels in the dark (48). A later study showed that the constitutive activity was caused by the F550I mutation at the pore region (49) raising the hypothesis that the constitutive activity of the Ca^2+^ permeable TRP channel leads to toxic increase in cellular Ca^2+^ and degeneration (48, 49, 50). Since MβCD suppressed both the LIC and the constitutive activity of TRP induced by metabolic inhibition (Fig. 3), it was interesting to examine whether MβCD suppresses the constitutive activity of the *trp*^*P365*^ mutant. Since the *trp*^*P365*^ mutant is characterized by extremely fast retinal degeneration, the patch clamp measurements were performed on a late-stage pupa (48). At this developmental stage the expression level of phototransduction components changes rapidly and because of the difficulty to determine the developmental stage accurately (48, 51) one expects variability in the measurements performed on different flies. As observed previously, current measurements in the dark of repeated voltage ramps (i-V curve) measured from *trp*^*P365*^ mutant photoreceptors revealed a large outward current at positive membrane voltages (Fig. 5*A*). These i-V curves are typical of light induced i-V curves measured in photoreceptors of the *trpl*^*302*^ mutant fly expressing WT TRP channels and thus confirmed that the TRP pore channel mutant is constitutively active (48). The i-V curves were relatively stable under sustained perfusion of standard extracellular solution for a duration of 130 s (Fig. 5*A*). Strikingly, application of MβCD in the dark suppressed the constitutive activity in photoreceptors of the *trp*^*P365*^ mutant in 8/11 tested flies. This suppression was manifested by a strong reduction of the current during repeated voltage ramps (i-V curve, Fig. 5, *B* and *C*). The observation that MβCD suppressed a constitutively active TRP mutant–channel, thus delimited MβCD suppression of TRP channel activity to the plasma membrane (see Discussion).

### MβCD did not affect activation of the TRP and TRPL channels by PUFAs

**Figure 6:**
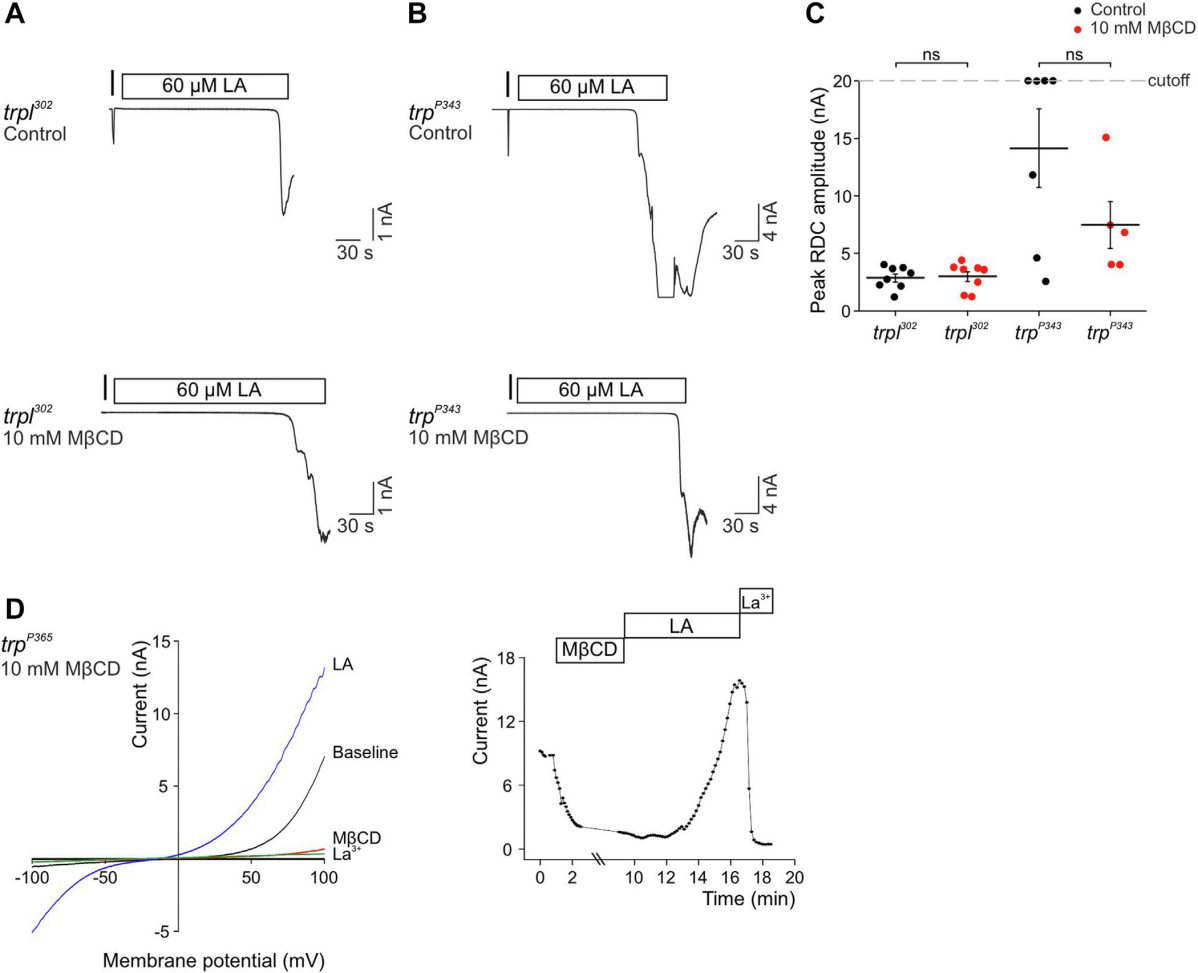
**MβCD that strongly suppressed the LIC did not affect the robust PUFA activated TRP and TRPL dependent currents.***A* and *B*, *top*: whole-cell patch clamp recordings from *trpl*^*302*^ (*A*, expressing TRP channels) and *trp*^*P343*^ (*B*) mutant ommatidia displaying activation of TRP (*A*) and TRPL (*B*) channels in the dark following application of 60 μM linoleic acid (LA, *rectangles*). A *short orange test light pulse* that elicited a short LIC is represented by a vertical bar. *Bottom*: whole-cell patch clamp recordings from *trpl*^*302*^ (*A*) and *trp*^*P343*^ (*B*) mutant ommatidia displaying activated TRP (*A*) and TRPL (*B*) channels in the dark following application of 60 μM LA (*rectangles*) in the presence of 10 mM MβCD. A *short orange test light pulse* is represented by a vertical bar. The light response of this test light was eliminated by the MβCD, which had no effect on the LA-induced activation of the TRP and TRPL channels. *C*, *scatter plot* displaying the peak amplitude of the currents resulting from channel activation in the dark following application of 60 μM LA to *trpl*^*302*^ (expressing TRP channels) and *trp*^*P343*^ (expressing TRPL channels) mutant photoreceptors, in the presence (*red*, n_*trpl302*_ = 8 n_*trpP343*_ = 5) or absence (*black*, n_*trpl302*_ = 8 n_*trpP343*_ = 7) of MβCD. Two-tailed Mann-Whitney *U* test was preformed (ns *p* > 0.05). *D*, *left:* representative i-V curves measured from isolated ommatidia of the *trp*^*P365*^ mutant, which express constitutively active TRP channel: *Baseline*, initial i-V curve showing the constitutive activity of the *trp*^*P365*^ photoreceptor. MβCD, i-V curve showing strong suppression of the constitutive activity of the *trp*^*P365*^ photoreceptor by MβCD. LA, i-V curve showing strong reactivation of the suppressed *trp*^*P365*^ current by application of LA. La^3+^, i-V curve showing maximal suppression of the current induced by La^3+^. *Right*: maximal current amplitudes measured from *trp*^*P365*^ mutant ommatidia in the dark as a function of time. The depicted current points show the initial constitutive *trp*^*P365*^ current and its gradual decline after application of 10 mM MβCD (*top rectangular*). Application of LA (60 μM, *top rectangular*) was followed by reactivation and even enhancement of the constitutive *trp*^*P365*^-induced TRP current. This current was strongly suppressed by application of La^3+^ (20 μM). The current points were measured at +140 mV holding voltage as a function of time. LIC, light-induced current; MβCD, methyl-β-cyclodextrin; TRP, transient receptor potential; TRPL, TRP-like; PUFA, poly unsaturated fatty acid.

Two main methods have been used to activate the natively expressed TRP and TRPL channels in a light-independent manner (*i.e.*, in the dark): (i) by inhibition of DAG kinase activity, either directly using the *rdgA* mutation or indirectly by depletion of ATP with mitochondrial uncouplers (Fig. 3). (ii) By application of PUFAs (22). One major difference between these two TRP/TRPL-dependent dark currents generation is that the CCCP (and *rdgA*)-induced currents are PLC-dependent (21, 52), while the PUFA-activated TRP/TRPL-dependent dark current is PLC-independent (25, 53). Since the TRP and TRPL channel activation by light or by ATP depletion was inhibited by MβCD, it was interesting to examine the effect of MβCD on the PUFA-activated TRP/TRPL-dependent currents. Importantly, we found that both the *trpl*^*302*^ photoreceptors (TRP channels, Fig. 6, *A* and *C*) and the *trp*^*P343*^ photoreceptors (TRPL channels, Fig. 6, *B* and *C*) were activated by PUFA (linoleic acid, LA) in the presence of MβCD (Fig. 6*C*). The reason for the observed long delay in PUFA activation of the TRP/TRPL channels is not known. This result was convincingly demonstrated by an initial suppression of constitutive TRP-dependent current of the *trp*^*P365*^ mutation by MβCD that was changed into current activation following addition of LA (Fig. 6*D*). These results indicate that suppression of the response to light by MβCD did not arise from damage to the light activated channels that remained active following PUFA activation in the presence of MβCD. The results further suggest that PUFA activate the TRP/TRPL channels by a mechanism that is different from the physiological activation of the channels that requires PLC.

### MβCD loaded with sterols suppressed the LIC while α-cyclodextrin revealed a much smaller effect on the LIC

**Supporting Figure S1:**
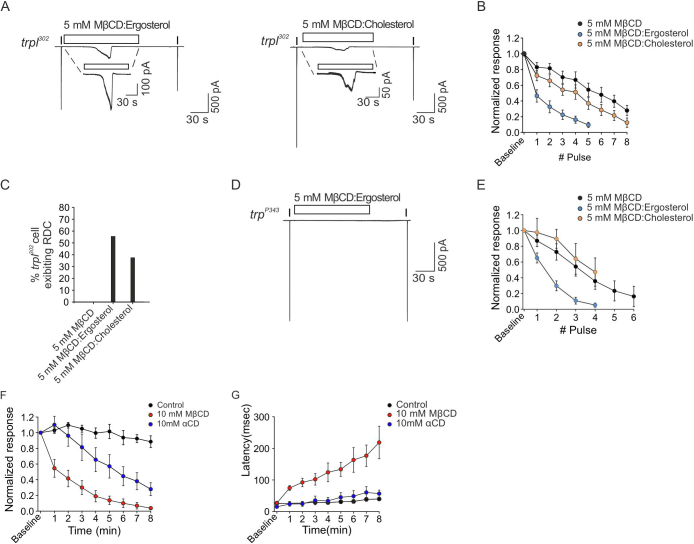
**MβCD loaded with either ergosterol or cholesterol suppressed the light induced current arising from activation of the TRP and TRPL channels.***A*, representative whole-cell patch clamp recordings displaying TRP channels activity in the dark (RDC) induced by application of MβCD (5 mM) loaded with either ergosterol (*left*) or cholesterol (*right*) in 40 to 50% of the recorded cells (see histogram in *panel C*). The LIC in response to a *short orange light pulses* are also shown before and after application of the tested solution. *B*, graphs presenting the normalized peak amplitude of the responses to repetitive *orange light pulses* as a function of time, recorded from *trpl*^*302*^mutant flies, in the presence of “empty” MβCD (5 mM) (*black*, n = 6) or MβCD (5 mM) loaded with either ergosterol (*green*) or cholesterol (*red*, n = 6). Each light response was normalized to the control LIC for each fly. Error bars are the SEM. *C*, histogram presenting the percentage of the *trpl*^*302*^ photoreceptor cells that were activated in the dark (RDC) in the presence of 5 mM MβCD:Ergosterol or 5 mM MβCD:Cholesterol. *D*, the LIC recorded from *trp*^*P343*^ mutant flies in response to a *short orange light pulses* are shown before and after application of MβCD (5 mM) loaded with ergosterol. *E*, graphs presenting the normalized peak amplitude of the responses to repetitive *orange light pulses* as a function of time, recorded from *trp*^*P343*^ mutant flies, in the presence of "empty" MβCD (5 mM) (*black*, n = 6) or MβCD (5 mM) loaded with either ergosterol (*green*) or cholesterol (*red*, n = 6). Each light response was normalized to the control LIC for each fly. Error bars are the SEM. *F*, graphs presenting the normalized peak amplitude of the responses to repetitive *orange light pulses* as a function of time, recorded from *trpl*^*302*^ photoreceptor cells, in the presence of SES (*black*, n = 6), 10 mM MβCD (*red*, n = 6), and 10 mM αCD (*blue*, n = 6). Each light response was normalized to the control LIC for each fly. Error bars are the SEM. *G*, graphs presenting the latency from the light onset to the beginning of the light response to repetitive *orange light* as a function of time, recorded from *trpl*^*302*^ photoreceptor cells, in the presence of SES (*black*, n = 6), 10 mM MβCD (*red*, n = 6), and 10 mM αCD (*blue*, n = 6). αCD, α-cyclodextrin; LIC, light-induced current; MβCD, methyl-β-cyclodextrin; RDC, rundown current; TRP, transient receptor potential; TRPL, TRP-like.

We also examined the effect of sterol addition to photoreceptor's membrane by incubating isolated *Drosophila trpl*^*302*^ and *trp*^*P343*^ ommatidia with MβCD-loaded with either ergosterol or cholesterol (Fig. S1). Application of MβCD loaded with either ergosterol or cholesterol gradually inhibited the responses to trains of light pulses, while the rate of response suppression by MβCD-loaded with ergosterol was faster than “empty” MβCD and MβCD loaded with cholesterol (Fig. S1, *B* and *E*). Interestingly, addition of MβCD loaded with ergosterol and to a lesser extent MβCD loaded with cholesterol, activated in several experiments the TRP (Fig. S1*A*) channels, but not the TRPL channels (Fig. S1*D*) in the dark. As a control for the specificity of MβCD in modulation of sterol levels relative to phospholipids, we incubated isolated *Drosophila trpl*^*302*^ ommatidia with α-cyclodextrin (αCD), since αCD was shown to sequester phospholipids and not sterols (1). Incubation of isolated *Drosophila trpl*^*302*^ ommatidia with αCD slowly reduced the peak amplitude of the LIC but after a large delay relative to MβCD and had a considerable smaller effect during αCD incubation (Fig. S1*F*). In addition, unlike MβCD, αCD had no effect on the response latency (Fig. S1*G*). These experiments suggest that rhabdomeral sterol or phospholipid content and presumably its spatial organization are important parameters in controlling channel activity by a still unclear mechanism (see Discussion).

## Discussion

In the present study, we used MβCD as a tool for investigating the effects of modulating ergosterol, the dominant sterol of *Drosophila* plasma membrane, on the phototransduction cascade. The dramatic, but selective, effects of ergosterol modulations on the activity of the TRP/TRPL channels, either when induced by light or during constitutive activation in the dark, together with measurements of TRP/TRPL currents simultaneously with light-induced hydrolysis of PIP_2_, enabled a new insight on the gating mechanism of the TRP/TRPL channels.

Phototransduction of *Drosophila* is a well-characterized cascade, enabling genetic dissection of ubiquitous physiological phenomena. It is also considered the fastest G-protein–mediated cascade (54) with tight kinetic parameters allowing quantitative analysis. Hence, *Drosophila* phototransduction allows dissecting the physiological effects of ergosterol modulation on a PLC-mediated cascade *ex vivo*. Incubation of isolated ommatidia with MβCD decreased the amplitude and increased the response latency of the LIC in time scales of minutes. Since, MβCD has been shown to sequester also PIP_2_ (1), but to a lesser extent compared with sterol sequestration, it is not clear whether the observed effects of MβCD arise, at least in part, from depletion of the PLC substrate PIP_2_. It is important to note that unlike some of the mammalian TRP channel, the *Drosophila* TRP/TRPL channels do not have PIP_2_-binding site and PIP_2_ has no regulatory role, except for being a substrate of PLC (55, 56). Three findings suggest that the observed effects of MβCD did not arise from depletion of PIP_2_: (i) the initial fluorescence intensity of Tb^R332H^-YFP that reports on PIP_2_ levels at the plasma membrane was similar in the absence and presence of MβCD (Fig. 4*F*). (ii) The light-induced translocation of Tb^R332H^-YFP was maintained in the presence of MβCD, while the LIC was virtually eliminated. (iii) Incubation with MβCD suppressed both quantum bump frequency and amplitude, with a more pronounced effect on quantum bump frequency. The observed reduction in bump frequency is not expected to result from PIP_2_ depletion. Accordingly, genetic modulation of light-induced PIP_2_ hydrolysis that was obtained by using weak and strong mutations of PLC was characterized by a large reduction in bump amplitude, while quantum efficiency of bump induction (a corelate of bump frequency) was largely unchanged (57). In conclusion, these combined results suggest that MβCD have only minor effect (if any) on the levels of the PLC substrate, PIP_2_ as measured by light-induced translocation of Tb^R332H^-YFP (Fig. 4). However, we observed that incubation of the photoreceptors with αCD (that is known to sequester mostly phospholipids (1), also decreased the light response amplitude (Fig. S1*F*), but has no effect on response latency (Fig. S1*G*). The apparent discrepancy with the above conclusion is readily resolved by results showing that the action of α-CD is mostly on phospholipids at the outer leaflet of the plasma membrane (58), leaving the PLC substrate, PIP_2_ at the inner leaflet relatively unchanged. Thus, the observed effects of α-CDs on the LIC did not arise from depletion of PIP_2_ but from a still unclear mechanism.

An important advance in the study of *Drosophila* phototransduction stemmed from the ability to randomly activate the TRP/TRPL channels in the dark without generation of quantum bumps (24, 37). There are several ways to obtain this dark activation of the channels: i) depletion of ATP (24) that presumably inhibits DAG kinase causing DAG accumulation, or by direct inhibition of DAG kinase, which is encoded by the *rdgA* gene (38), *via rdgA* mutations (21). ii) Activation of the TRP/TRPL by PUFA application (22). iii) By the F550I mutation at the pore region of the TRP channel (48, 49). Interestingly, all modes of TRP/TRPL channel activation either by light or in the dark, which are PLC-dependent are also blocked by application of MβCD. In contrast, channel activation by PUFA is neither PLC-dependent nor suppressed by MβCD suggesting that: (i) MβCD does not operate by nonspecific irreversible inactivation of the channels due to damage to the channels and (ii) MβCD suppression of PLC-dependent channel activation is likely operating on the physiological PLC-mediated TRP/TRPL channel activation.

A critical result of this study was obtained by simultaneous recordings of the LIC and translocation of Tb^R332H^-YFP florescence as a reliable measure of the dynamic PLC hydrolyzing activity during light. After 20 to 30 min of incubation with MβCD, we observed >100-folds' suppression of the LIC without similar suppression of Tb^R332H^-YFP translocation (Fig. 4). This result indicates that while strongly suppressing TRP/TRPL channels activity, MβCD neither depleted PIP_2_ and abolished PLC activity nor inhibited the activity of the signaling proteins upstream to PLC (*i.e.*, the photopigment and G_q_). This conclusion was supported by the observation that MβCD suppressed a constitutively active TRP mutant–channel, *trp*^*P365*^, suggesting that TRP channel activity is a target of MβCD action. Together, we were able for the first time, to dissociate between PLC and TRP/TRPL channel activation *ex vivo*, by monitoring light induced PLC activity when channel activity was highly attenuated. The implication of this observation is that rhabdomeral PIP_2_ pool and PLC activation by light are resistant to ergosterol reduction.

A strong support for the notion that “empty” MβCD actually reduces ergosterol level in the photoreceptors came from studies of *Drosophila* dietary modulations. A noninvasive method of reducing membrane ergosterol content in *Drosophila*, without application of MβCD to dissociated ommatidia was achieved by feeding flies with ezetimibe (59). Ezetimibe is a sterol-lowering drug used in the treatment of hypercholesterolemia. It can reduce mammalian plasma cholesterol levels by blocking uptake through the small intestine, but does not stop cellular synthesis of cholesterol (60). Specifically, it acts to block Niemann-Pick C1-like 1 protein, responsible for transfer of cholesterol across the gut wall. The *Drosophila* homolog of this protein, npc1b, has been shown to regulate absorption of sterol across the midgut in flies (61). Interestingly, studies have suggested that sterol trafficking mechanisms in insects and vertebrates are highly conserved (62). A. S. Randall (59) found that feeding *Drosophila* with ezetimibe, strongly affected the amplitude and waveform of the LIC measured in isolated ommatidia, in a similar manner to incubation with MβCD. Although their tested flies were raised on a diet with reduced PUFA content, the effect of reduced PUFA content in isolation was much smaller than the effects of either incubation of isolated ommatidia with MβCD or testing isolated ommatidia of *Drosophila* fed with ezetimibe. Together, feeding *Drosophila* with ezetimibe revealed effects similar to those of MβCD on the LIC, suggesting that the major effect of MβCD on the light response is mediated by sequestration of ergosterol (59). However, localization of the effects of ergosterol reduction in the phototransduction cascade was not investigated in these studies.

In light of the above findings, one may ask for a possible mechanism by which ergosterol sequestration uncouples PLC activation by light from TRP/TRPL channel gating. A clue to a possible answer came from studies showing that PIP_2_ forms clusters (PIP_2_ cluster) separated from lipid rafts in the plasma membrane (9). Accordingly, the ergosterol-resistant PIP_2_ pool found in this study may form such PIP_2_ cluster. The DRM lipid rafts domain that was disrupted by dietary restriction of ergosterol intake in *Drosophila* photoreceptors, which contains the TRP channel and PLC attached to the scaffold protein INAD (28) *via* separate PDZ domains (63) may form a distinct plasma membrane nanoscopic lipid domain (11). According to this view, lateral movement of the signaling proteins between nanoscopic lipid domains (*i.e.*, lipid rafts and PIP_2_ clusters (11)) may generate the light response in a still unclear way, while ergosterol reduction/addition (Fig. S1, *A*, *B*, and *E*) disrupts this movement and inhibits channels gating. Hence, ergosterol-dependent movement of the signaling proteins between nanoscopic lipid domains may account for some of the observed effects of MβCD (9, 10, 11, 64).

## Experimental procedures

### Fly stocks

The following strains and mutants of *Drosophila melanogaster* were used: *;trpl*^*302*^*,cn,bw;, ;;trp*^*P343*^*, ;;p[rh1-Tb*^*R332H*^*-YFP,w*^*+*^*]* (46)*, ;;trp*^*P365*^.

Flies were raised at 24 °C in a 12 h light/dark cycle on standard corn meal food. Pupae vials were wrapped with aluminum foil 12 h before eclosion. Newly eclosed flies were used. *trp*^*P365*^ mutant flies, were raised only in the dark, and pupae at ∼90 h post puparium stage were used (65).

### Light stimulation

A xenon high-pressure lamp (Lambda LS, Sutter Instruments) was used, and the light stimuli were delivered to the ommatidia by means of epi-illumination *via* the objective lens (*in situ*). The effective intensity of the orange light (Schott OG 590 edge filter) at the specimen was calibrated using a bioassay, by measuring the rate of single photon responses (bumps) from WT flies at normal Ca^2+^ conditions (effective photons per second, as previously described (36)).

### Electrophysiology

Whole-cell recording was performed at 21 °C using borosilicate patch pipettes of 8 to 12 MΩ resistance, an Axopatch 1D (Molecular Devices) voltage-clamp amplifier, Digidata 1440A, and pClamp software (https://support.moleculardevices.com/s/article/Axon-pCLAMP-11-Electrophysiology-Data-Acquisition-Analysis-Software-Download-Page) (Molecular Devices). Series resistance values were <25 MΩ and were routinely compensated to >80% when recording macroscopic responses >100 pA, but not when recording bumps (as previously described (36)).

### Bump detection

Bumps were detected offline using the event detection threshold search function of pClamp 10.2.0.14 software (Molecular Devices). The following parameters were used: trigger, 3 pA; re-arm, 2 pA; pre-trigger, 1 ms; post-trigger, 1 ms; and minimum allowed duration, 10 ms as previously described (36).

### Imaging of dissociated ommatidia

Fluorescence from tagged isolated ommatidia was viewed with an Olympus UPlanFI 60× NA 1.30 oil immersion objective on an Olympus inverted microscope, using freshly dissociated ommatidia. Images were captured at two frames/sec, using a CCD camera (Andor). The average intensity was measured in ROIs in the rhabdomere and cytosol offline using NIS Elements AR imaging software (https://nis-elements-viewer.software.informer.com/4.2/https://www.nisoftware.net/NikonSaleApplication/). Region of interest was manually determined.

### Solutions

Standard extracellular solution (SES) contained (mM): 120 NaCl, 5 KCl, 4 MgCl_2_, 10 TES, 25 Proline, 5 alanine 1.5 CaCl_2_, pH 7.15.

MβCD solution contained extracellular solution with 10 mM MβCD.

CCCP solution contained extracellular solution with 10 mM CCCP.

LA solution contained SES (without 1.5 mM CaCl_2_) solution with 60 μM LA.

Intracellular solution contained (mM) 140 potassium gluconate, 2 MgCl_2_, 10 TES, 4 ATP magnesium salt, 0.4 GTP sodium salt, 1 β-NAD, pH 7.15.

Cholesterol/ergosterol enrichment: for the detailed procedure of solution preparation see (3). Briefly, cholesterol or ergosterol were dissolved in 1:1 methanol:chloroform (cholesterol) or chloroform (ergosterol) to generate 50 mg/ml stock solution. The stock solution was added to a glass tube, the solvent was evaporated, and 5 mM MβCD (in SES, without CaCl_2_) was added to the dried cholesterol/ergosterol.

The tube was sonicated for 10 min, placed in a shaking incubator overnight at 37 °C, and was filtered using 0.45 mm filter. The solution was kept in room temperature for 6 days.

## Data availability

All data are contained within the manuscript.

## Supporting information

This article contains supporting information.

## Conflict of interest

The authors declare that they have no conflict of interests with the contents of this article.

## Supporting information

Supporting Figure S2 **A video showing the light-induced PIP**_**2**_**hydrolysis by PLC as monitored by Tb**^**R332H**^**-YFP fluorescence translocation from the rhabdomere to the cytosol in response to intense blue (488 nm) light in the presence (*right*) and the absence (*left*) of 10 mM MβCD.** The video represents Tb^R332H^-YFP fluorescence translocation of the photoreceptors of Figure 4*A*. MβCD, methyl-β-cyclodextrin; PIP_2_, phosphatidylinositol 4,5-bisphosphate; PLC, phospholipase C.
